# Oxidative stress in *Prymnesium parvum*: cellular mechanisms, redox regulation, and implications for harmful algal bloom management

**DOI:** 10.3389/fpls.2026.1812273

**Published:** 2026-04-22

**Authors:** Tomasz Krupnik

**Affiliations:** Department of Molecular Plant Physiology, Institute of Environmental Biology, Faculty of Biology, University of Warsaw, Warsaw, Poland

**Keywords:** hydrogen peroxide (H2O2), mitochondrial reactive oxygen species, osmotic stress, peroxiredoxins, photorespiration, programmed cell death, prymnesins, reactive oxygen species (ROS)

## Abstract

*Prymnesium parvum* is a mixotrophic haptophyte known for its harmful algal blooms (HABs), which are driven by its ability to adapt to fluctuating environmental conditions, particularly osmotic and oxidative stress. This work explores the physiological, molecular, and ecological responses of *P. parvum* to osmotic shifts, both hypo- and hyperosmotic—and their intersection with mechanisms of oxidative stress. Rapid adjustments in cell volume, membrane composition, and cytoskeletal structure, as well as biosynthesis of compatible solutes, allow *P. parvum* to tolerate a wide salinity range (0.5–30 PSU). Gene expression studies reveal up-regulation of aquaporins, ion transporters, and stress-related chaperones, orchestrated by signaling cascades involving calcium and reactive oxygen species (ROS). Although it lacks catalase and possibly conventional photorespiration, *P. parvum* maintains ROS homeostasis through peroxiredoxins and superoxide dismutase. Importantly, oxidative stress, salinity, and nutrient status influence prymnesin toxin production, underscoring the ecological relevance of these stress responses.

## 1 Oxidative stress in *P. parvum*

Oxidative stress significantly influences *P. parvum*, particularly under highsalinity, nutrient limitation, or fluctuating light conditions. As in other photosynthetic organisms, ROS are generated during both photosynthetic electron transport and mitochondrial metabolism. Importantly, cellular ROS levels reflect a dynamic balance between their production and scavenging rather than simple accumulation. Moreover, ROS exhibit a dual role, acting as damaging agents under stress conditions while simultaneously functioning as signaling molecules that regulate adaptive physiological responses. Due to its toxic and mixotrophic lifestyle, *P. parvum* appears to have evolved distinctive oxidative stress responses that are tightly integrated with its survival strategies. The apparent absence of canonical catalase activity suggests that this alga relies on an alternative antioxidant system. Indeed, oxidative and osmotic stress responses are closely interconnected, as salinity-induced cellular imbalance enhances ROS generation (Rezayian et al., 2019). Antioxidant defenses, particularly superoxide dismutase (SOD) and peroxiredoxins, play a central role in ROS detoxification (Hounslow et al., 2021). Notably, the lack of catalase may be associated with a reduced photorespiratory capacity, a pathway typically responsible for substantial H_2_O_2_ production. This characteristic may contribute to the pronounced sensitivity of *P. parvum* to relatively low H_2_O_2_ concentrations ~0.3–0.4 mM (~10–15 ppm) and highlights a potential vulnerability that can be exploited in strategies aimed at controlling harmful bloom events.

## 2 ROS Generation

**Figure 1:**
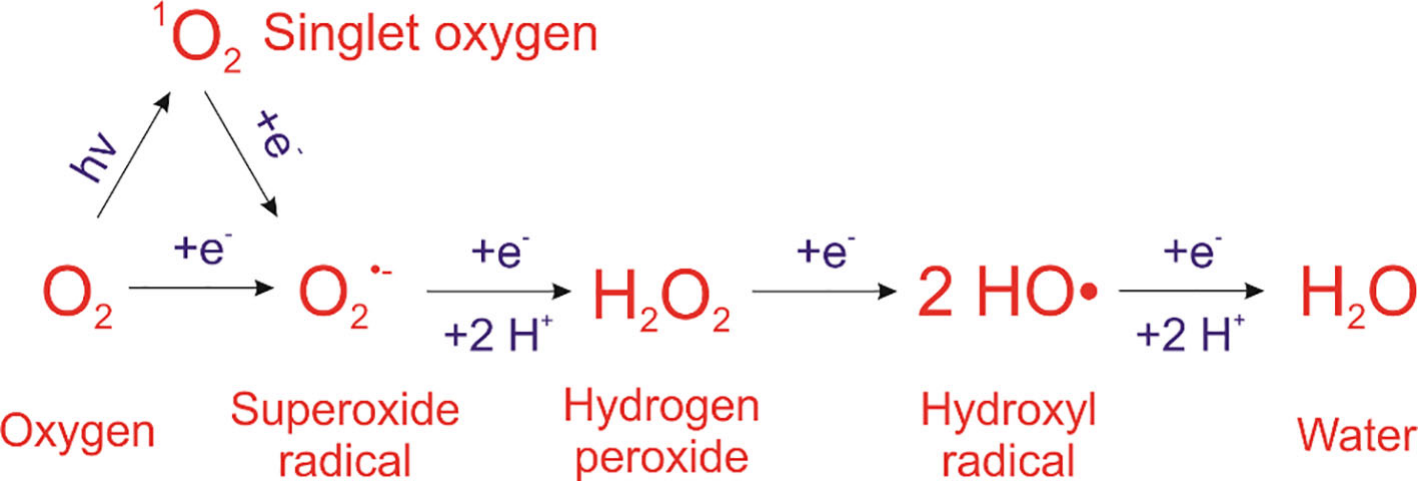
Reactive oxygen species (ROS) produced in a sequential one-electron reduction from oxygen to water.

ROS such as superoxide, hydrogen peroxide (H_2_O_2_), and hydroxyl radicals (•OH) are primarily produced in Photosystem I under high light or CO_2_ limitation. PSII can also contribute when electron flow is disrupted (Murata et al., 2007). ROS reactivity follows the order: •OH > ¹O_2_ > H_2_O_2_ > •O_2_^-^ (Apel and Hirt, 2004; Schweitzer and Schmidt, 2003; **Figure 1**). Accumulated ROS cause lipid peroxidation, protein oxidation, and nucleic acid damage, and at sufficiently high concentration, leads to cell death. Quantitative assessments of ROS production have been achieved in model systems, particularly in isolated organelles where rates of H_2_O_2_ emission have been be directly measured, with reported values on the order of 0.3–6.7 nmol min^-^¹ mg protein^-^¹ depending on substrate conditions and preparation (Starkov, 2010). In microalgae such as *Chlamydomonas reinhardtii*, ROS accumulation can also be quantified in a concentration-dependent manner under stress conditions (Almeida et al., 2017) as intracellular content or fluorescence signal rather than organelle-specific flux. However, such compartment-specific and quantitative analyses remain largely unavailable for *P. parvum*, representing a key gap in current understanding.

### 2.1 Photosynthesis-associated ROS production

Photosynthesis in algae is a dominant source of ROS under both normal and stress conditions (Vineeth et al., 2023). The thylakoid membranes, where the photosynthetic electron transport chain (PETC) resides, are particularly prone to ROS generation because of their high metabolic activity, presence of electron carriers, and exposure to molecular oxygen.

#### 2.1.1 Photosystem II and Singlet Oxygen generation

In PSII, when excitation exceeds the rate of photochemical quenching, especially under conditionsof excessive light or limited nutrients, triplet-state chlorophyll (^3^Chl*) forms.^3^Chl* readily reacts with ground-state molecular oxygen (^3^O_2_) togenerate singlet oxygen (^1^O_2_), a highly electrophilic ROS molecules (Triantaphylidès and Havaux, 2009). The excited-state molecule ^1^O_2_ is generated predominantly at the reaction center of PSII and, to a lesser extent, in the light harvesting complexes (LHCs) (Krieger-Liszkay, 2005), and it is the main ROS responsible for photooxidative damage in plants (Triantaphylides et al., 2008). In species such as *Chlamydomonas reinhardtii*, exposure to high intensity light (e.g., > 600 µmol photons m^-^² s^-^¹) increases singlet oxygen production, which in turn can oxidize key PSII proteins (like D1), lipids in the thylakoid membrane, and pigments such as β-carotene. The xanthophyll cycle (involving violaxanthin, antheraxanthin, and zeaxanthin) and nonphotochemical quenching (NPQ) help protect PSII from overexcitation by dissipating excess energy as heat (Niyogi, 1999; Triantaphylidès and Havaux, 2009). Carotenoids are essential not only for photoprotection but also for direct quenching of ^1^O_2_. However, when these mechanisms are overwhelmed, irreversible damage to PSII ensues, leading to photoinhibition. Damaged D1 protein must be degraded and resynthesized, a process that is also light dependent and sensitive to ROS.

#### 2.1.2 Photosystem I and superoxide production via the Mehler reaction

Under conditions where NADP^+^ regeneration is limited—such as during high light exposure or when carbon assimilation is constrained (e.g., under nutrient or iron limitation)—photosystem I (PSI) can transfer electrons to molecular oxygen instead of NADP^+^. This process, known as the Mehler reaction, involves the reduction of O_2_ to the superoxide anion (• O_2_^-^), primarily via ferredoxin (Asada, 2006). Nutrient stress or fluctuating high light can transiently or periodically deplete downstream electron acceptors, causing electrons to be diverted to oxygen and resulting in substantial accumulation of •O_2_^-^ (Tjus et al., 2001). Superoxide is rapidly dismutated by superoxide dismutase (SOD) into hydrogen peroxide (H_2_O_2_), which is relatively more stable and can diffuse through membranes. While this reaction may prevent over-reduction of PSI, it introduces the risk of oxidative damage to stromal proteins and nucleic acids if H_2_O_2_ accumulates beyond the capacity of scavenging enzymes like ascorbate peroxidase (APX) or catalase (CAT).

#### 2.1.3 Cyclic electron flow and its dual role in ROS homeostasis

Cyclic electron flow (CEF) around PSI allows electrons from ferredoxin to re-enter the plastoquinone (PQ) pool via PGR5/PGRL1 or NDH pathways, generating a proton gradient for ATP synthesis without NADPH production. This is critical to meeting cellular ATP demands during nitrogen assimilation, carbon concentration mechanisms, and photorespiration (Shikanai, 2014). In algae, CEF can be rapidly activated under high light or CO_2_-limiting conditions, thereby preventing over-reduction of PSI. However, excessive or dysregulated CEF can promote overreduction of the PQ pool, leading to backflow of electrons to O_2_ via the plastid terminal oxidase (PTOX) or direct PQ-mediated reduction of O_2_, generating ROS (Munekage et al., 2002). Thus, while CEF is a protective process, it must be fine-tuned to avoid enhancing chloroplastic ROS production. Similarly, preillumination seems to activate the CEF (Shi et al., 2021), thus enhancing its PSI protective activity. In mutants or conditions with defective NPQ or Calvin cycle activity, CEF may paradoxically exacerbate oxidative stress.

### 2.2 Mitochondrial ROS production

The mitochondria of algae play a role in ATP production during cellular respiration. They also function as redox hubs, contributing significantly to intracellular ROS pools.

#### 2.2.1 Electron transport chain and ROS generation

The mitochondrial electron transport chain (ETC) comprises complexes I–IV and alternative oxidase. During aerobic respiration, Complexes I (NADH:ubiquinone oxidoreductase) and III (cytochrome bc_1_ complex) are the main sites of superoxide (•O_2_^-^) generation (Zhao et al., 2019). Electron leakage occurs when upstream carriers are over-reduced, leading to premature transfer of electrons to O_2_ and generation of •O_2_^-^ (Nolfi-Donegan et al., 2020; Murphy, 2009). The rate of •O_2_^^-^^ production from the electron transport chain (ETC) depends on the concentration of theone-electron donor at a specific site and the rate at which this redox-active donor reacts withmolecular oxygen (O_2_) and can be modulated by local O_2_ concentration,Δp, electron flux, and ATPase activity (Nolfi-Doneganet al., 2020). In photosynthetic eukaryotes, including algae and higher plants, ROSproduction is further modulated by metabolic interactions between mitochondria and chloroplasts. During high light exposure or nutrient stress, increased photorespiration and reductant overflow from the chloroplast can increase mitochondrial electron input, increasing the probability of electron leakage and ROS formation (Bailleul et al., 2015; Noguchi and Yoshida, 2008). In particular, glycine oxidation during photorespiration contributes significantly to mitochondrial ROS levels (Foyer & Noctor, 2003). In algal species such as *Phaeodactylum tricornutum* and *Haematococcus pluvialis*, metabolic shifts under nitrogen starvation or light-dark transitions can significantly increase mitochondrial ROS. Alternative oxidase (AOX) in some algae can bypass Complex III and IV, reducing ROS output by maintaining electron flow under stress. .

Major sites of ROS generation in the mitochondrial electron transport chain (ETC) are depicted. Superoxide (•O_2_^-^) is the primary ROS formed, predominantly in Complexes I and III, but also at several other redox-active enzymes. The red arrows indicate the side of the mitochondrial inner membrane where ROS are released. Within the matrix, superoxide is rapidly converted to hydrogen peroxide (H_2_O_2_) by manganese superoxide dismutase (MnSOD), while in the intermembrane space, this function is carried out by copper/zinc SOD (Cu/ZnSOD). The matrix also contains antioxidant systems, including the glutathione peroxidase (GPx)/glutathione reductase (GR) cycle, which detoxifies H_2_O_2_. ROS-producing components shown in red:

1. Complex I (NADH:ubiquinone oxidoreductase);
2. Complex II (succinate dehydrogenase);
3. mGPDH (mitochondrial glycerol-3-phosphate dehydrogenase);
4. ETF/ETFQO (electron transfer flavoprotein and its oxidoreductase);
5. DHODH (dihydroorotate dehydrogenase);
6. Complex III (cytochrome bc_1_ complex)

### 2.3 Mitochondrial and chloroplast ROS in programmed cell death

**Figure 2:**
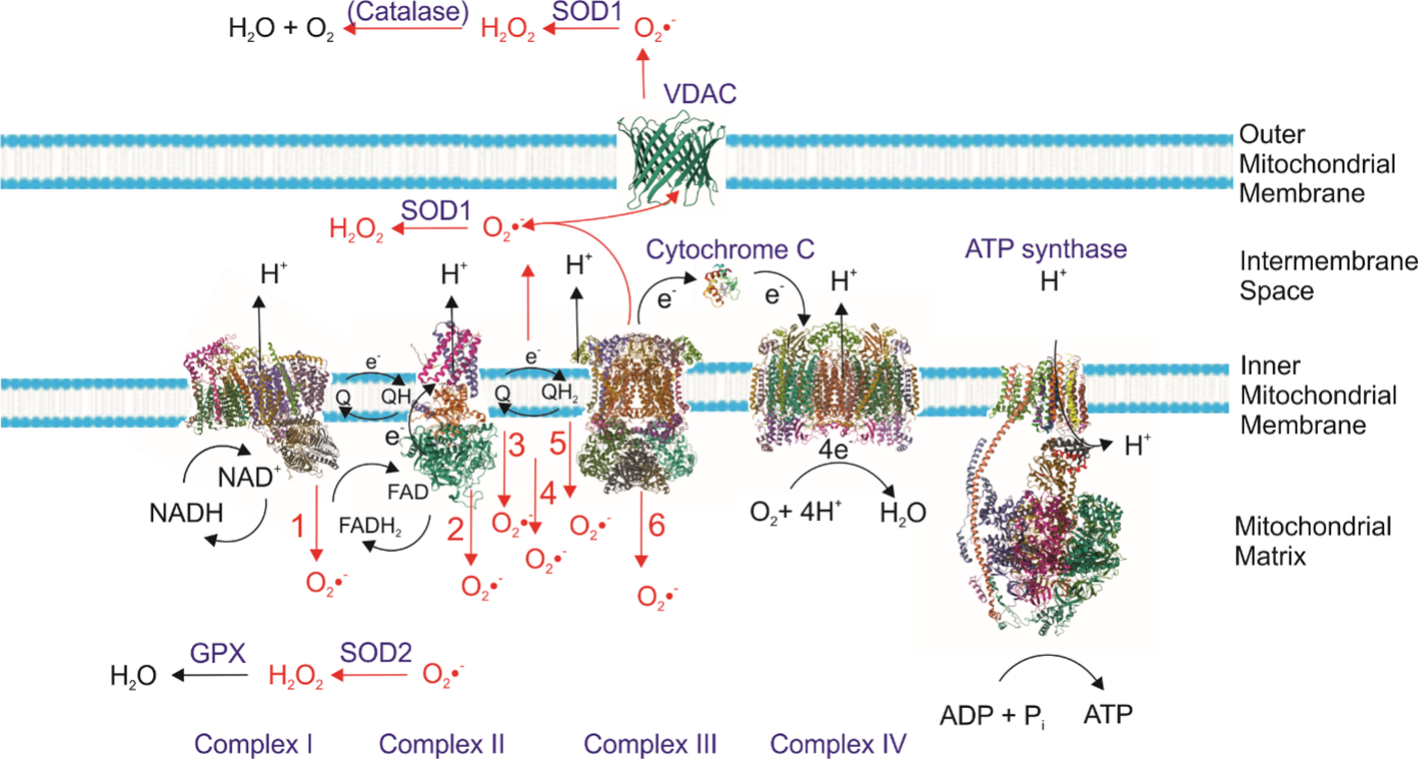
Sites of mitochondrial reactive oxygen species (ROS) production.

The programmed cell death (PCD) has been extensively elucidated and characterized in animals;however, a comparable understanding in plants remains limited (Ambastha et al., 2015; **Figure 2**). In fact, plant PCD research is still in its infancy, with progress marked by sporadicreports and isolated studies. Elevated mitochondrial ROS production is a key trigger for PCD inalgae (Bai et al., 2023; Ning et al., 2002). This process is characterized by chromatin condensation,externalization of phosphatidylserine, DNA fragmentation, and caspase-like activity (Xu et al., 2022). In bloom-forming haptophytes such as*Emiliania huxleyi*, viral infection leads to ROS-mediated PCD that regulates bloomtermination (Bidle et al., 2007). Algal mitochondriapossess multiple redox sensors and antioxidant enzymes (Mn-SOD, peroxiredoxins, and glutathioneperoxidases) that regulate redox status and prevent inadvertent ROS-triggered death. However, onceoxidative thresholds are crossed, mitochondrial dysfunction accelerates cell death. Photosyntheticreactions and the reduced pool of plastoquinone are the main sources of ROS in the chloroplast(Van Breusegem and Dat, 2006; Xing et al., 2013; Zapata et al.,2005) and light-induced oxidative stress is the main factor inducing chloroplast-programmedcell death. Chloroplast PCD can be triggered by biotic interactions involving the chlorophyll catabolic protein Accelerated Cell Death 2 (ACD2), which is typically localized in the chloroplast (Yao and Greenberg, 2006; Pattanayak et al., 2012). ROS have been shown to trigger heat-induced apoptosis (Doyle et al., 2010) in *Arabidopsis spp*, and recently, the role of EXECUTER protein (EX1, EX2) was shown to be essential in the response of plants to stress induced by single oxygen (^1^O_2_) (Zhang et al., 2014). Multiple proteins encoded by ROS-responsive genes have been shown to participate in signaling pathways leading to the hypersensitive response (HR) and consequent cell death (Landoni et al., 2013). An important, nonphotosynthetic source of ROS in chloroplast is the coversion of protochlorophyllide (Pchlide) to chlorophyllide (Chlide) that takes place in the presence of light. Both dark adaptation as well as herbicide ALA (5-aminolevulinic acid) cause an accumulation of Mg-tetrapyrroles. These, when exposed to light, begin to generate singlet oxygen (^1^O_2_) (Tripathy and Oelmuller, 2012).

### 2.4 Peroxisomal and enzymatic ROS generation

**Figure 3:**
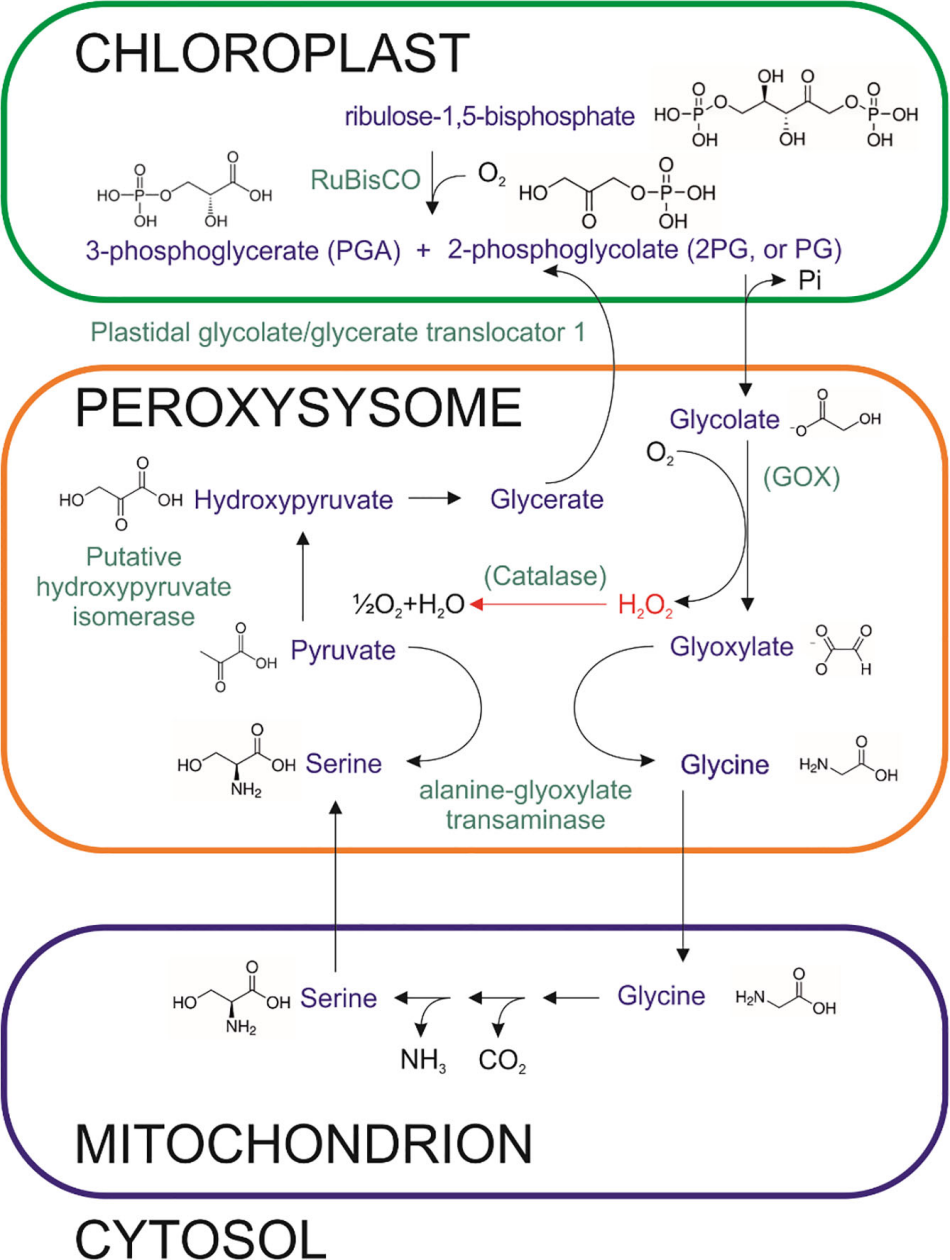
Schematic view of the photorespiratory pathway in *P. parvum*. Candidate proteins in *P. parvum* were identified by BLAST analysis. Enzymes are shown in green font. Hypothetical proteins are shown in parentheses. The accession numbers are (Plastidal glycolate/glycerate translocator 1, chloroplastic A0AB34KBF0, A0AB34J8H0; alanine--glyoxylate transaminase A0AB34JE10; Putative hydroxypyruvate isomerase A0AB34IUM7; phosphoglycerate dehydrogenase, AB1Y20_009476; phosphoserine transaminase A0AB34K3R0, Rubisco, ribulose-1,5-bisphosphate Q9GFX2_PRYPA).

Peroxisomes play a crucial role in fatty acid metabolism and photorespiration in algal cells andare major sources of hydrogen peroxide (H_2_O_2_) due to their oxidative enzymatic machinery. Plant peroxisomes contain numerous enzymes capable of generating H_2_O_2_, including glycolate oxidase (GOX), acyl-CoA oxidase (ACOX), urate oxidase (UO), polyamine oxidase, copper amine oxidase (CuAO), sulfite oxidase (SO), sarcosine oxidase (SOX), and superoxide dismutase (SOD) (Hauck et al., 2014; Corpas et al., 2017; **Figure 3**). The H_2_O_2_ produced in peroxisomes can diffuse into the cytosol,acting as a signaling molecule that modulates stress responses and gene expression (Foyer and Noctor, 2003; Koletti et al, 2025). However, excessive H_2_O_2_ accumulation can lead to oxidative damage to proteins, lipids, and nucleic acids, highlighting the need for tight regulation by catalase and other antioxidant systems. In algal cells, peroxisomal ROS production is particularly important under high-light or nutrient-stress conditions, linking metabolic activity with cellular redox balance. In addition to peroxisomal sources, enzymatic ROS production also occurs in the cytoplasm and the plasma membrane, contributing further complexity to cellular redox networks (Mandal et al., 2022; Zheng et al., 2025).

#### 2.4.1 H_2_O_2_ production during photorespiration

Photorespiration in plants is traditionally considered a wasteful process because it reduces the efficiency of carbon assimilation. Glycolate, a key photorespiratory metabolite, is derived from 2-phosphoglycolate, a by-product of RuBisCO’s oxygenase activity (**Figure 3**). Although RuBisCO primarily catalyzes CO_2_ fixation during photosynthesis, its oxygenase activity increases when the CO_2_/O_2_ ratio decreases (Giordano et al., 2005). Glycolate is subsequently converted to glyoxylate and then to glycine, which is further metabolized to serine, linking photorespiration to nitrogen metabolism and providing intermediates for cellular redox balancing and reactive oxygen species (ROS) detoxification (**Figure 3**). These conversions occur across multiple subcellular compartments: glycolate is oxidized in peroxisomes, glycine decarboxylation and serine formation occur in mitochondria, and several recycling steps take place in the chloroplast, illustrating the tight integration of photorespiration with cellular metabolism and ROS signaling (Noctor and Foyer, 2016).

Photorespiration also contributes to the generation of reactive oxygen species (ROS),particularly in peroxisomes. Enzymes such as glycolate oxidase (GOX) produce hydrogen peroxide(H_2_O_2_) during the conversion of glycolate to glyoxylate. Furthermore,increased metabolic activity within the peroxisomes and mitochondria during photorespirationenhances ROS generation. In most photosynthetic organisms, H_2_O_2_ is detoxifiedby catalase within peroxisomes. However, *Prymnesium parvum* does not possess a catalase gene, suggesting that it has probably evolved alternative strategies to limit photorespiratory flux or mitigate ROS accumulation. In many algae, especially under high O_2_ or low CO_2_ conditions, RuBisCO oxygenase activity triggers photorespiration. The resulting 2-phosphoglycolate enters the glycolate pathway, where GOX converts glycolate into glyoxylate, releasing H_2_O_2_ as a by-product (Foyer and Noctor, 2005b). This H_2_O_2_ is typically detoxified by peroxisomal catalase or transported to the cytoplasm, where it can function as a redox signal or contribute to oxidative stress. In rice (*Oryza sativa*), the GOX–catalase complex dynamically associates and dissociates to fine-tune peroxisomal H_2_O_2_ levels (Noctor et al., 2002; Zhang et al., 2016). The associated state helps maintain peroxide homeostasis. Under stress conditions, such as those mediated by salicylic acid (SA), this complex dissociates, leading to inhibition of catalase and elevated H_2_O_2_ levels (Zhang et al., 2016; Kohli et al., 2019). Other proteins known to influence the activity of the GOX–catalase complex include the γb viral protein (Yang et al., 2018) and the small heat shock protein Hsp17.6CII in *Arabidopsis* (Li et al., 2017), both of which modulate H_2_O_2_ accumulation and oxidative stress. *Prymnesium parvum* relies primarily on the Calvin–Benson–Bassham (CBB) cycle for carbon fixation. Because RuBisCO can also catalyze oxygenation reactions, the organism may possess at least a partial photorespiratory pathway similar to those described in higher plants and green algae, potentially involving enzymes such as glycolate oxidase–like proteins and serine hydroxymethyltransferase (SHMT) (Hagemann et al., 2016). However, genome-wide and transcriptome analyses performed in BLAST for *P. parvum* suggest that this pathway is incomplete. Previous work of Rahman et al. (2014) suggest contribution from photorespiration in *P. parvum* in conditions of Fe stress, which may indicate presence of photorespiratory pathways with possibly different enzymes. Specifically, *P. parvum* lacks clear homologs for both the catalase and *GOX* genes, as confirmed by the absence of such sequences in the UniProt database. Many algae utilize glycolate dehydrogenase (GYD) or related enzymes that transfer electrons to alternative acceptors instead of molecular oxygen. Such pathways do not generate H_2_O_2_ directly and therefore may explain the apparent absence of both GOX and catalase in *P. parvum*. This raises the possibility of a noncanonical, compartmentalized, or truncated photorespiratory mechanism, adapted to its unique ecological niche.

Within the phylum Haptophyta, catalase genes are present in only four species, with Phaeocystisantarctica being the sole representative possessing a catalase–peroxidase (KatG) enzymesuperfamily, annotated under UniProt ID: A0A7S0NGX7. BLAST searches in *P. parvum*revealed 37 hits grouped into 17 hypothetical proteins, all lacking functional assignment butshowing partial homology to the KatG superfamily of catalase–peroxidase. The E-values of these hits ranged from 2 × 10^-^²^6^ to 7 × 10^-^¹^4^, with sequence coverage between 25.05% and 30.48%. Representative gene IDs include AB1Y20_004181, AB1Y20_022242, and AB1Y20_003996. However, none of these candidates could be confidently assigned as canonical catalase homologs based on sequence similarity criteria. These observations are consistent with the currently available genome resource *for P. parvum* (Kuhl et al., 2024). It should be noted, however, that the absence of detectable homologs does not exclude the presence of enzymes with catalase-like activity, as functionally analogous proteins with low sequence similarity or convergent enzymatic function may exist. Similarly, only six species within the Haptophyta encode enzymes related to glycolate metabolism. In particular, *Chrysochromulina tobinii* is the only species with an annotated glycolate oxidase gene (*gox*; UniProt ID: A0A0M0JA87). In *P. parvum*, two genes were identified that encode putative glycolate transporters (UniProt IDs: A0AB34J8H0, A0AB34KBF0). BLAST analysis detected three candidate GOX-related sequences in *P. parvum*, grouped into two clusters of hypothetical proteins (gene IDs: KAL1499766.1, KAL1499767.1, and KAL1520742.1). These hits exhibited strong homology to the LldD superfamily (FMN-dependent α-hydroxy acid oxidases), with E-values ranging from 1 × 10^-^¹³³ to 6 × 10^-^³⁴ and sequence coverage between 28.53% and 48.51%. This superfamily includes enzymes that catalyze the oxidation of 2-hydroxy acids to 2-oxo acids, suggesting that these proteins may perform glycolate oxidase–like activity in *P. parvum*. However, no canonical GOX homologs were identified based on sequence similarity, consistent with the currently available genome resource (Kuhl et al., 2024). These findings suggest that glycolate metabolism in *P. parvum* may rely on functionally analogous enzymes with divergent sequence features and potentially distinct electron acceptors.

Members of the LldD superfamily typically function as flavin-dependent oxidoreductases that transfer electrons to alternative acceptors such as quinones rather than directly to molecular oxygen. Consequently, these enzymes do not necessarily produce hydrogen peroxide, which may partly explain why catalase genes are absent from the *P. parvum* genome.

Although once considered metabolically wasteful, photorespiration and the associated hydrogenperoxide (H_2_O_2_) flux are now recognized as adaptive mechanisms that contributeto the regulation of cellular energy balance and redox signaling, particularly under fluctuatinglight intensities or limited carbon availability. In addition to its primary role in recoveringcarbon from the oxygenation reaction, photorespiration may protect against over-reduction andacceptor limitations in the photosynthetic electron transport chain (Heber and Krause, 1980; Kozaki and Takeba,1996; Takahashi et al., 2007). This essentialfunction is underscored by the existence of mutants with a high-carbon-requirement (HCR) phenotype,which are unable to grow under ambient CO_2_ conditions but remain viable at elevatedCO_2_ levels (Somerville, 2001). Photorespirationfirst evolved in cyanobacteria and has been conserved in existing algae and land plants (Eisenhut et al., 2006; Bauweet al., 2010; Kern et al., 2013). Itspersistence is likely driven by the historical increase in atmospheric oxygen, which improvesRuBisCO oxygenase activity and leads to the accumulation of toxic intermediates such as2-phosphoglycolate (2-PG) (Husic et al., 1987; Norman and Colman, 1991). Over evolutionary time, selection favored the use of glycolate oxidase (GOX) over glycolate dehydrogenase (GlcD) to convert glycolate to glyoxylate in photorespiratory metabolism (Kehlenbeck et al., 1995; Bauwe et al., 2010). Although GOX generates H_2_O_2_ as a byproduct, its higher maximal catalytic activity (V_max_) in oxygen-rich environments, together with its close functional coupling to catalase-mediated detoxification, makes it a highly efficient pathway. Consequently, the presence of GOX is widely regarded a hallmark of plant-type photorespiration (Hagemann et al., 2013; Kehlenbeck et al., 1995).

## 3 Fatty acid β-oxidation and H_2_O_2_ accumulation

During β-oxidation in peroxisomes, acyl-CoA oxidases catalyze the initial dehydrogenationstep, transferring electrons to O_2_ and eventually forming H_2_O_2_(Ghisla and Thorpe, 2004). This is particularly active duringlipid turnover in algae under starvation, desiccation, or transition to resting stages (e.g., cystsin *Dunaliella* spp. or akinetes in cyanobacteria). In *P. parvum*, fatty acid β-oxidation likely occurs predominantly in peroxisomes, as in other stramenopiles and haptophytes (two groups of single-celled eukaryotic algae (Petersen et al., 2014), that are evolutionarily related, both having acquired their plastids from a red alga). In general, the process involves stepwise removal of two-carbon units from fatty acyl-CoA molecules, producing acetyl-CoA, FADH_2_, and NADH. Because acetyl-CoA cannot cross organelle membranes directly, it is typically converted into transportable intermediates such as citrate or acetate, which can be shuttled to mitochondria and subsequently re-converted to acetyl-CoA for entry into central metabolism. This metabolic coupling links peroxisomal β-oxidation to cellular redox homeostasis, as the generation of NADH and H_2_O_2_ can alter intracellular redox balance and modulate ROS-dependent signaling pathways (Foyer and Noctor, 2005a). Acetyl-CoA then enters the tricarboxylic acid (TCA) cycle or contributes to the glyoxylate shunt under stress conditions (Li-Beisson et al., 2019; Masclaux-Daubresse et al., 2020). In particular, the first step—catalyzed by acyl-CoA oxidase—produces H_2_O_2_ directly by transferring electrons to molecular oxygen rather than via the electron transport chain, making β-oxidation a significant source of ROS (Quijano et al., 2016). Environmental stressors such as high salinity, nutrient depletion, or light stress upregulate lipid catabolism in *P. parvum* (Liu et al., 2024). This response mobilizes triacylglycerols stored in the lipid bodies, leading to enhanced β-oxidation. In Cyanobacteria, transcriptomic evidence suggests increased expression of genes encoding oxidases and catalases under salt stress, although these processes occur in the cytosol due to the absence of peroxisomes (Edvardsen et al., 2016; Granéli and Salomon, 2010), suggesting a compensatory antioxidant response. The resulting H_2_O_2_, generally, can be compartmentalized or detoxified by catalase, but excessive production may leak into the cytoplasm, affecting nuclear gene expression and cytosolic enzymes through redox modifications (Martemucci et al., 2022). In *P. parvum*, the rise in intracellular H_2_O_2_ levels can act as a signaling molecule, triggering defense responses and possibly upregulating prymnesin biosynthesis (Woźnica et al., 2024).

## 4 NADPH oxidases and oxidative bursts

NADPH respiratory burst oxidases (RBOHs) are membrane-bound enzymes that generate superoxide bytransferring electrons from NADPH to molecular oxygen. RBOHs are key components of defenseresponses, stress signaling, and development in multicellular plants (Kim et al., 2025). In algae, functional RBOH homologs have been identified inspecies such as *Chara corallina*, *Chlamydomonas reinhardtii*, and *Prymnesium parvum* (Anderson et al., 2016; Anderson et al., 2011). These enzymes are activated by calcium influx, phosphorylation, and small GTPases in response to environmental stimuli such as hypersalinity, pathogen contact, or mechanical stress (Liu et al., 2020). In harmful algal bloom (HAB) species, respiratory burst oxidase homolog (RBOH) activity may contribute to toxin release and allelopathic interactions by altering the surrounding redox environment (Hervé et al., 2006). ROS generated by NADPH oxidases can further activate downstream signaling pathways, including mitogen-activated protein (MAP) kinases, nitric oxide (NO) signaling, and phytohormone-like regulators, establishing redox-based feedback loops that are essential for algal stress responses (Lesser, 2006). These mechanisms highlight the dual role of ROS in HAB species as both signaling molecules and modulators of intercellular interactions under stress conditions.

## 5 Antioxidant defense

### 5.1 Enzymatic response to ROS

The antioxidant system comprises both enzymatic and non-enzymatic components (Gauthier et al., 2020; Rezayian et al., 2019). Enzymatic antioxidants include superoxide dismutase(SOD), which converts superoxide to H_2_O_2_, as well as catalases, ascorbate peroxidases, and glutathione reductase (Al-Rashed et al., 2016). Interestingly, *Prymnesium parvum* appears to lack a catalase and GOX enzymes, possibly due to a limited or alternatively arranged antioxidant machinery (Wisecaver et al., 2023). However, it possesses two SOD genes (A0AB34JJ92, A0AB34JCU8) and five peroxiredoxin genes (A0AB34ISF7, A0AB34JEZ0, A0AB34JK80, A0AB34K7A3, A0AB34JNI0), which may contribute to ROS detoxification (Coulombier et al., 2021). Given the apparent absence of catalase in *P. parvum*, peroxiredoxins likely represent a primary enzymatic mechanism for H_2_O_2_ detoxification, supported by superoxide dismutase activity and glutathione-based redox systems. Non-enzymatic antioxidants, ascorbate, flavonoids, carotenoids, tocopherols, glutathione, and phenolic compounds, quench ROS and prevent oxidative chain reactions (Mittler, 2002; Foyer & Noctor, 2005 and 2008; Gauthier et al., 2020). Cellular repair mechanisms also play a key role in the restoration of oxidized lipids, proteins, and DNA (Møller et al., 2007; Noctor et al., 2014).

### 5.2 Physiological responses to osmotic stress

Osmotic shock occurs when abrupt changes in external salinity lead to water influx or efflux,disrupting osmotic equilibrium. Hypoosmotic stress (from freshwater inflow) can cause cell swellingor lysis, while hyperosmotic stress (from increased salinity) can result in dehydration or metabolicarrest (Kirst, 1990; Brand, 1984). The survival of *P. parvum* in a wide salinity range (0.5–30 PSU; Mazur-Marzec et al., 2025; Baker et al., 2007; Zhao et al., 2009) reflects its biochemical and genetic capacity to mitigate osmotic stress. Significant levels of salinity change (about 0.5 PSU) were found to cause significant release of toxins and eventually cell death (Woźnica et al., 2024). Osmotic imbalance can also enhance reactive oxygen species (ROS) production by disrupting ion homeostasis and membrane integrity, which impairs electron transport processes in chloroplasts and mitochondria (Lesser, 2006; Rezayian et al., 2019). In addition, rapid changes in salinity may activate NADPH oxidase (RBOH)-dependent ROS generation as part of stress signaling, linking osmotic perception to redox-regulated responses (Lesser, 2006; Kim et al., 2025). Under osmotic stress, cells upregulate aquaporins, osmolyte transporters, and enzymes for the biosynthesis of compatible solutes such as proline, trehalose, and glycine betaine (Szabados and Savouré, 2010; Elbein et al., 2003). Consequently, *P. parvum* thrives across a broad salinity range after an adaptation period. An immediate response of *P. parvum* cell was observed to increase its size and assume a more spherical shape (Woźnica et al., 2024).

### 5.3 Cell volume regulation and membrane and cytoskeletal remodeling

*Prymnesium parvum* inhabits dynamic aquatic environments where salinity can fluctuate rapidly, particularly in estuarine and brackish ecosystems. To survive such osmotic shifts, the alga must precisely regulate cell volume and maintain structural integrity. This is achieved through tightly coordinated ion transport, osmolyte accumulation, membrane remodeling, and cytoskeletal reorganization. During hypoosmotic stress, when external salinity suddenly decreases, water influx increases due to the osmotic gradient, risking cellular swelling and rupture. *P. parvum* responds by rapidly reducing internal osmolarity through the active efflux of inorganic ions such as Na^+^, K^+^, and Cl^-^ (Woźnica et al., 2024). This process is mediated by a suite of ion channels and transporters, including voltage-gated, mechanosensitive, and Ca²^+^-dependent ion channels that detect membrane tension and transduce stress signals (Shetty et al., 2019). Calcium (Ca²^+^) ions act as a primary second messenger in osmotic stress signaling, linking membrane-level stress perception to downstream activation of MAPK and CDPK pathways. Transient increases in cytosolic Ca²^+^ are likely triggered by mechanosensitive and ion channels, coordinating redox signaling and transcriptional responses (Brooks et al., 2010; Caron et al., 2023; Dodd et al., 2010).

Similar mechanisms are known in other euryhaline microalgae, suggesting a conserved strategyamong phytoplankton. Under hyper-osmotic stress, water tends to leave the cell, causing shrinkageand potential plasmolysis. To counteract this, *P. parvum* accumulates compatiblesolutes, small organic molecules that stabilize proteins and membranes without interfering withcellular metabolism. The main osmolytes identified in marine microalgae includedimethylsulfoniopropionate (DMSP), proline, glycine betaine, and polyols (Karsten et al., 1991; Stefels, 2000).These molecules function not only as osmoprotectants but also as antioxidants and signalingmolecules under stress conditions (Renzetti et al.,2025). Membrane fluidity is another critical parameter affected by salinity changes (Guo et al., 2022). Changes in osmotic pressure alter lipid packing and membrane phase behavior, which can affect transport, receptor function, and signal propagation. To maintain optimal membrane function, *P. parvum* likely modulates its lipid composition, including the saturation level of fatty acids and the content of sterols and phospholipids (Thompson, 1996). It also produces omega-3 and omega-6 PUFAs (e.g., docosahexaenoic acid, DHA), which enhance membrane fluidity and flexibility (Carballeira et al., 2001). Similar adaptive changes have been reported in other microalgae and halotolerant species, such as *Dunaliella salina* (GusChina and Harwood, 2006). The cytoskeleton, particularly the actin network, plays a central role in maintaining the cellular architecture under osmotic stress. Actin filaments dynamically reorganize in response to changes in turgor pressure and membrane curvature, helping to preserve cell shape and facilitate vesicle trafficking and organelle positioning (Liu Y. et al., 2015). In *P. parvum*, cytoskeletal remodeling may be essential for redistributing ion transporters, reorganizing chloroplast positioning, or directing the trafficking of stress-response proteins. Actin involvement in stress signaling is supported by studies in other algal and plant systems, where actin dynamics is regulated by Ca²^+^ signaling and reactive oxygen species (ROS) (Zulu et al., 2018).

### 5.4 Molecular responses and gene regulation

Transcriptomic analyses show that osmotic stress upregulates genes for ion transporters (e.g.,Na^+^/H^+^ antiporters), aquaporins, osmoprotectant biosynthesis, and molecularchaperones (Liu Z. et al., 2015; Talarski et al., 2016). Stress perception is likely mediated by calcium signaling and ROS accumulation, which activate MAPK and CDPK signaling cascades (Brooks et al., 2010; Caron et al., 2023). Heat shock proteins (e.g., HSP70, HSP90) and antioxidative enzymes (e.g., superoxide dismutase, glutathione peroxidase) are also upregulated under salinity stress, suggesting a conserved response to oxidative damage (Maki et al., 2008; Sithtisarn et al., 2017; Castielli et al., 2009).

## 6 Link between ROS and toxin production

Toxin biosynthesis in *Prymnesium parvum* is metabolically demanding andassociated with elevated mitochondrial and plastid activity, resulting in increased production ofreactive oxygen species (ROS), including superoxide, hydrogen peroxide, and hydroxyl radicals.Beyond being by-products of oxidative metabolism, ROS function as signaling molecules and mayregulate prymnesin biosynthesis (Manning and La Claire, 2010; Freitag et al., 2011). ROS-dependent signaling pathways, including those mediated by NADPH oxidases and redox-sensitive kinases, may directly modulate gene expression and enzymatic steps involved in toxin biosynthesis, linking oxidative stress to secondary metabolism (Lesser, 2006; Kim et al., 2025). Moreover, ROS accumulation may act as a physiological trigger that shifts cellular metabolism toward defensive and allelopathic strategies, promoting prymnesin production under stress conditions (Lesser, 2006; Caron et al., 2023). Environmental stressors such as salinity fluctuations, nutrient limitation, extreme temperatures, and high light intensities enhance ROS accumulation and stimulate toxin production (Graniéli et al., 2012; Weissbach and Legrand, 2012; Caron et al., 2023). These factors can act synergistically, promoting oxidative imbalance and activating secondary metabolism as an adaptive response.

Prymnesin production is typically induced under stress conditions and during trophic shifts fromphotoautotrophy to mixotrophy or phagotrophy, where toxins may facilitate prey digestion.Structurally, prymnesins exhibit detergent-like properties due to their polyether rings and multiplehydroxyl groups, supporting their role in membrane disruption (Rasmussen et al., 2016). Due to their detergent-like structure, prymnesins are highly ichthyotoxic, causing damage to gill epithelia and impairing ion regulation and gas exchange in fish.

## 7 Susceptibility of *Prymnesium parvum* to hydrogen peroxide

The susceptibility of *Prymnesium parvum* to hydrogen peroxide(H_2_O_2_) is influenced by several factors, including the applied concentration,the duration of exposure, the physiological condition of the cells, and environmental parameterssuch as salinity and light intensity. Laboratory studies have shown that *P. parvum*is sensitive to relatively low concentrations of H_2_O_2_, typically ranging from~30 to 300 µM (1–10 mg L^-^¹; 1–10 ppm), depending on the exposuretime, which is usually measured in hours. At these levels, H_2_O_2_ inducesoxidative stress by damaging cell membranes and photosynthetic machinery, leading to reducedphotosynthetic efficiency and cell viability (Moreno-Andréset al., 2023; Weenink et al., 2015). Hydrogen peroxide naturally decomposes into water and oxygen through chemical and biological redox reactions. This degradation typically occurs over several hours to a few days, depending on biological activity and the presence of redox-active metals such as iron and manganese (Cooper and Zepp, 1990; Häkkinen et al., 2004). Importantly, H_2_O_2_ is inherently unstable in aqueous environments, and its effective concentration can decline rapidly due to photodecomposition, catalytic breakdown by transition metals, and reactions with dissolved organic matter. As a result, at environmentally relevant concentrations (<100 µM), the biological impact of H_2_O_2_ is often governed by short-term exposure immediately after application rather than prolonged steady-state levels (Cooper and Zepp, 1990; Häkkinen et al., 2004). In natural freshwater systems, H_2_O_2_ concentrations range from 30 to 900 nM (1–30 µg L^-^¹; 1–30 ppb) (Cooper et al., 1989; Häkkinen et al., 2004). These background concentrations are maintained not by persistence of H_2_O_2_, but by a dynamic balance between continuous photochemical and biological production and rapid degradation processes, resulting in a steady-state equilibrium. In the presence of redox-active metals such as Fe²^+^ or Mn²^+^ and exposed to light, hydrogen peroxide can undergo the Fenton reaction, generating highly reactive hydroxyl radicals (•OH), which contribute to oxidative damage by attacking cellular macromolecules such as lipids, proteins, and DNA (Zepp et al., 1992; Mittler, 2002; Apel and Hirt, 2004; Latifi et al., 2009).

Early work by Barroin and Feuillade (1986) showed thateven a low concentration of ~51 µM H_2_O_2_ (1.75 mg L^-^¹;1.75 ppm) significantly harmed cultures of the cyanobacterium *Planktothrixrubescens*, while a tenfold higher dose caused no observable damage to the green alga*Pandorina morum*. In aquaculture, Rach et al. (1997) used H_2_O_2_ at concentrations ranging from 4.3 mM to 213 mM H_2_O_2_ (145–7250 mg L^-^¹; 100–5000 (v/v) ppm; 100–5000 µL L^-^¹ used) to disinfect fish eggs from algal and fungal contaminants and found that juvenile fish tolerated up to ~43 mM H_2_O_2_ (1000 (v/v) ppm). Similarly, Wood et al. (2021) reported only a minor reduction in hypoxia tolerance in Atlantic salmon after a 20-minute exposure to ~54 mM H_2_O_2_ (1250 ppm H_2_O_2_) at 12 °C. Field applications of hydrogen peroxide for the mitigation of harmful algae blooms (HAB) vary widely. The UK protocol by Wagstaff et al. (2021) applied 1.2 to 1.5 mM (40–50 mg L^-^¹; 40–50 ppm) and achieved suppression of *P. parvum* proliferation for up to 96 hours, after which the population returned to pretreatment levels, suggesting transient effects with limited long-term ecological impact. In contrast, the Texas Parks and Wildlife Department protocol (Neisch et al., 2012; based on Southard, 2005) reported effective lysis of *P. parvum* cells at ~1.8 to 14.7 mM (62.5–500 mg L^-^¹) within 24 hours, ~92 mM (3125 mg L^-^¹) in one hour, and ~368 mM (12,500 mg L^-^¹) in just 15 minutes. While higher concentrations improve efficacy, they also increase the risk of collateral damage, particularly to sensitive life stages such as fish eggs.

Cyanobacteria such as *Microcystis* and *Raphidiopsis* show acutesensitivity to H_2_O_2_, with lethal thresholds of ~100 µM (3.4 mgL^-^¹) and ~50 µM (1.7 mg L^-^¹), respectively (Drábková et al., 2007a; Matthijs et al., 2012). This increased vulnerability is partly due to their lack of robust antioxidant enzyme systems (Miller et al., 2000) and the anatomical exposure of their phycobilisomes, which are crucial for photosynthesis (Drábková et al., 2007b). Consequently, attempts to control *P. parvum* blooms with H_2_O_2_ can inadvertently result in the elimination of cyanobacterial biodiversity (Santos et al., 2021; Weenink et al., 2022). A recent large-scale application in Poland exemplifies the practical use of this method. Between 5 and 9 July 2024, the Polish Ministry of Climate and Environment treated the Kłodnica River with H_2_O_2_ at concentrations ranging from ~0.29 to 0.44 mM (10–15 mg L^-^¹; 10–15 ppm), achieving a reported 90–95% reduction in *P. parvum* abundance with only minor and short-lived ecological disruption (Weber et al., 2024).

Salinity may play a critical role in modulating the sensitivity of *P. parvum* toH_2_O_2_. Under low-salinity conditions (freshwater to mildly brackish), algal growth becomes slower and cells appear more bloated, making them more susceptible to oxidative damage, likely due to compounded osmotic stress causing membrane tension, which may compromise membrane integrity and reduce the efficiency of antioxidant defenses (Graneli et al., 2012; Sobieraj and Metelski, 2023; Woźnica et al., 2024). This physiological state likely enhances susceptibility to oxidative damage, making H_2_O_2_ particularly effective as a mitigation strategy in freshwater-influenced brackish environments where osmotic and oxidative stresses act synergistically. In contrast, moderate salinities can activate protective mechanisms, such as increased expression of antioxidant enzymes like catalase and peroxidase, which enhance the organism’s ability to detoxify H_2_O_2_ and limit cellular damage (Weissbach and Legrand, 2012).

Other natural intracellular processes like photosynthesis, photorespiration, respiration, andperoxisomal activity can lead to local increases in H_2_O_2_ concentration,exacerbating the deleterious effects on cell vitality. From a management perspective,H_2_O_2_ presents multiple advantages: it rapidly decomposes into non-toxicbyproducts, is effective at relatively low concentrations, and can selectively target HAB-formingspecies like *P. parvum* when applied with care (Southard, 2005; Pedersen and Pedersen,2012; Wagstaff et al., 2021). Nevertheless, precise dosing and ongoing environmental monitoring are essential to avoid negative impacts on nontarget phytoplankton and aquatic fauna. Furthermore, the effectiveness of H_2_O_2_ can be enhanced when combined with other stressors that compromise cellular ROS-scavenging systems, potentially allowing lower and safer dosages (Moreno-Andrés et al., 2023). To optimize treatment strategies, a deeper understanding of *P. parvum’s* oxidative stress response mechanisms is needed to identify physiological vulnerabilities that can be exploited in the field.
